# Attraction is altered via modulation of the medial prefrontal cortex without explicit knowledge

**DOI:** 10.3389/fnhum.2024.1333733

**Published:** 2024-08-14

**Authors:** Samantha Zorns, Claudia Sierzputowski, Sydney Ash, Molly Skowron, Anthony Minervini, Adriana LaVarco, Matthew Pardillo, Julian Paul Keenan

**Affiliations:** Cognitive Neuroimaging Laboratory, Montclair State University, Montclair, NJ, United States

**Keywords:** attractiveness, TMS, rTMS, appearance, MPFC

## Abstract

Previous studies have demonstrated that brain stimulation can alter an individual's physical appearance via dysregulation of the medial prefrontal cortex (MPFC). In this study, we attempted to determine if individuals who receive repetitive transcranial magnetic stimulation (rTMS) delivered to the MPFC were rated as more attractive by others. It has been previously reported that 1 hertz (Hz) (inhibitory) TMS can alter one's facial expressions such that frontal cortex inhibition can increase expressiveness. These alterations, detected by external observation, remain below the level of awareness of the subject itself. In Phase I, subjects (*N* = 10) received MPFC rTMS and had their photographs taken after each of the five stimulation conditions, in addition to making self-ratings across a number of variables, including attractiveness. In Phase II, participants (*N* = 430) rated five pictures of each of the Phase 1 individuals on attractiveness. It was found that there were no significant differences in self-assessment following rTMS (Phase I). However, attractiveness ratings differed significantly in Phase II. There was a significant difference found between 10 Hz TMS delivered to the MPFC (*p* < 0.001), such that individuals were rated as less attractive. Furthermore, 1 Hz TMS to the MPFC increased the number of ‘Most Attractive' ratings, while 10Hz TMS decreased the number of ‘Most Attractive' ratings (*p* < 0.001). These results suggest that the MPFC plays a role in attractiveness ratings to others. These data also support research showing that one's appearance can be altered below the level of awareness via rTMS. To our knowledge, this is the first investigation to examine how brain stimulation influences one's attractiveness.

## 1 Introduction

Initial attraction refers to a series of positive reactions toward a person, usually upon immediate encounter in which no close relationship has been established yet (Gerlach and Reinhard, 2018). There are several different types of positive reactions toward a person that comprise attraction: positive thoughts and beliefs, positive feelings and emotions, motivation to approach the person, and behavioral reactions such as getting physically closer to the person (Yuan et al., 2021; Baxter et al., 2022; Ueda, 2022).

Numerous studies have attempted to determine what makes a person attractive to others; for example, self-confidence (Shipman and Mumford, 2011) has been well-studied and known to have a significant impact on an individual's attractiveness (Li et al., 2020). It has been suggested that individuals who are perceived as possessing high levels of self-esteem may be assumed to possess other characteristics believed to be associated with high self-esteem, therefore signaling that the individual is worthy of pursuing as a mate (Zeigler-Hill and Myers, 2011). However, it is important to note that it is possible to be *overconfident* to the extent that attractiveness diminishes, and it was also noted that the link between attractiveness and confidence varied based on gender and sexuality (Zeigler-Hill and Myers, 2011).

The medial prefrontal cortex (MPFC) is important in goal-directed behavior; it constitutes value in order to achieve the highest reward (Miller, 2000; Laskowski et al., 2016), as well as social planning and self-insight (Barrios et al., 2008; Amati et al., 2010; Mattavelli et al., 2011). In humans, the MPFC has been identified as a critical region of self-worth (Bang and Fleming, 2018), suggesting that altering MPFC activity should directly influence a person's self-worth and possibly attractiveness.

Furthermore, stimulation of MPFC across a variety of methods can result in dysregulation in distal regions (Regenold et al., 2022). Specifically, repetitive transcranial magnetic stimulation (rTMS) delivered in an inhibitory manner causes direct suppression of the ventral striatal cortex, resulting in significant circuit-wide changes (Popa et al., 2019). It is well-demonstrated that suppression of MPFC, experimentally or otherwise, causes widespread ‘freeing' of one's regulated processes, networks, and circuits (Stuss and Alexander, 2000; Stuss et al., 2005; Stefano et al., 2013; Butler and Chiong, 2019; Leisman and Melillo, 2022; Nikolaidis et al., 2022).

rTMS is a non-invasive stimulation method used to either excite or inhibit different regions of the brain for a short period of time (Pascual-Leone et al., 1999; Maeda et al., 2000a,b; Chail et al., 2018; Shelansky et al., 2022). rTMS alters neural function, which is critical in determining causal relationships (George et al., 2003; Rossi et al., 2009; Demirtas-Tatlidede et al., 2010; Burke et al., 2019). To utilize rTMS, a coil is placed on the scalp, which produces electric pulses that create a magnetic field and modulate the excitability of the cortex (Mann and Malhi, 2022). Brain regions or any given circuit's casual role in cognitive, affective, or perceptual processes can be ascertained via TMS (Heinisch et al., 2011). To excite the cortical regions, 10 hertz (Hz) rTMS is used, while 1Hz rTMS decreases cortical activity and is thus seen as inhibitory (Yan et al., 2017; Begemann et al., 2020; De Risio et al., 2020; Che et al., 2021).

rTMS frequently targets the MPFC due to its involvement in social observation, self-awareness, and self-enhancement (Taylor-Lillquist et al., 2020; Duran et al., 2021). Previous studies demonstrated that exciting the MPFC using rTMS increases the amount of self-enhancement in an individual, while inhibiting the MPFC reduces the amount of self-enhancement (Kwan et al., 2007; Barrios et al., 2008; Amati et al., 2010; Luber et al., 2012; Shelansky et al., 2022; Yasin et al., 2022).

The MPFC is involved in most aspects of social perception and cognition (Cacioppo et al., 2012). Findings from Hutcherson et al. (2015) indicated that fostering social connections attracts a significant portion of the self-related MPFC. Furthermore, as a result of TMS in the MPFC, people tend to perceive faces as more trustworthy when positive visual primes are present before them rather than negative ones (Ferrari et al., 2017).

There is a lack of research regarding how others perceive a person who has undergone TMS. The current study aims to explore the level of attraction one experiences to a person who has undergone TMS to the MPFC in both an excitatory and inhibitory manner. Building on studies that have found a linkage between attraction and the MPFC, our study will extend previous research by employing both 10 Hz and 1Hz rTMS applied to the MPFC. We hypothesize that individuals will be rated as more attractive following inhibition of the MPFC. This study is unique in that we are altering the brain regions of the person being judged. Furthermore, by directly manipulating brain regions and thus moving beyond correlation, we can directly use participants as their own controls, allowing for more precise comparisons of attraction.

Changes induced by direct brain manipulation are often imperceptible. Lawson et al. reported that preferences for items in a forced-choice task were altered via rTMS, and participants were unaware that such influences existed (Lawson et al., 2023). In fact, rTMS can be used to induce cortical blindness such that conscious perception of a stimulus is not reported, even though it clearly influences performance (Boyer et al., 2005). We therefore assessed our simulated participants to determine if they noticed any changes in their own attractiveness. We assume that only outside observers would notice changes.

Karmann et al. (2016) found direct evidence that rTMS delivered to the MPFC resulted in changes that were externally noticeable yet imperceptible to the subjects themselves. Employing a study based on pain, the authors delivered uncomfortable stimuli to the subjects after either sham (no rTMS) or 1Hz rTMS (which inhibited the MPFC). It was found that no subjective differences in pain were reported. However, when the facial expressions of the subjects were recorded and analyzed, significant differences emerged. Participants were more expressive as rated using the facial action coding system (FACS) in their pain following inhibition of the MPFC without awareness of such differences.

The current study is novel in a number of ways. First, unlike the Karmann study, facial changes were rated by an extremely large sample (*N* = 430) rather than an automated system. Second, this study is the first to directly assess attractiveness following rTMS. Furthermore, we also examined the effects of cortical excitation rather than just inhibition. Individuals are highly inaccurate at self-assessment of attractiveness (Kościński, 2011; Greitemeyer, 2020). Therefore, we predicted a mismatch between self-reports of attractiveness and external evaluations.

## 2 Methods

### 2.1 Participants

For Phase I, a total of 10 participants were recruited for the study through flyers posted on the campus of Montclair State University, social media, and word of mouth. The participants were between the ages of 18 and 25 years old, three identified as male and seven as female. The participants were recruited through flyers and social media and offered $25 for their involvement in the experiment. The participants were treated ethically in accordance with American Psychiatric Association guidelines and appropriately screened for receiving TMS (Rossi et al., 2009; Groppa et al., 2012), and the study was approved through Montclair IRB (MSU-IRB-FY21-22-2595).

For Phase II, 430 participants were recruited through SONA, a standard online recruiting system that allows students to participate in research studies for course credit. The courses for this study were two introductory psychology classes. Participation in this study (or other studies) or an alternative assignment, was a course requirement. The entire duration of Phase I was ~3 weeks, while Phase II was ~3 months.

### 2.2 Materials

For the participants undergoing TMS, a Magstim 200 quick stimulator and a 7-cm figure-of-eight coil were utilized to provide pulses at 10 Hz and 1 Hz for all TMS stimulation. All presentations were made using Testable on a Lenovo ThinkPad T490. Trigno wireless MEP amplifiers and DelSys software were used to determine motor threshold (MT). For the duration of the experiment, the participants wore Lycra swim caps as well as earplugs (Wassermann, 1998; Lerner et al., 2019)

Two online platforms were used for the experiment: SONA (Sona-system.com) scheduling software and Testable (Testable.org) presentation software. Photographs were taken using an iPhone 7 at the highest resolution setting. All data were analyzed using standard PCs using SPSS 25.

### 2.3 Stimuli

**Figure 1:**
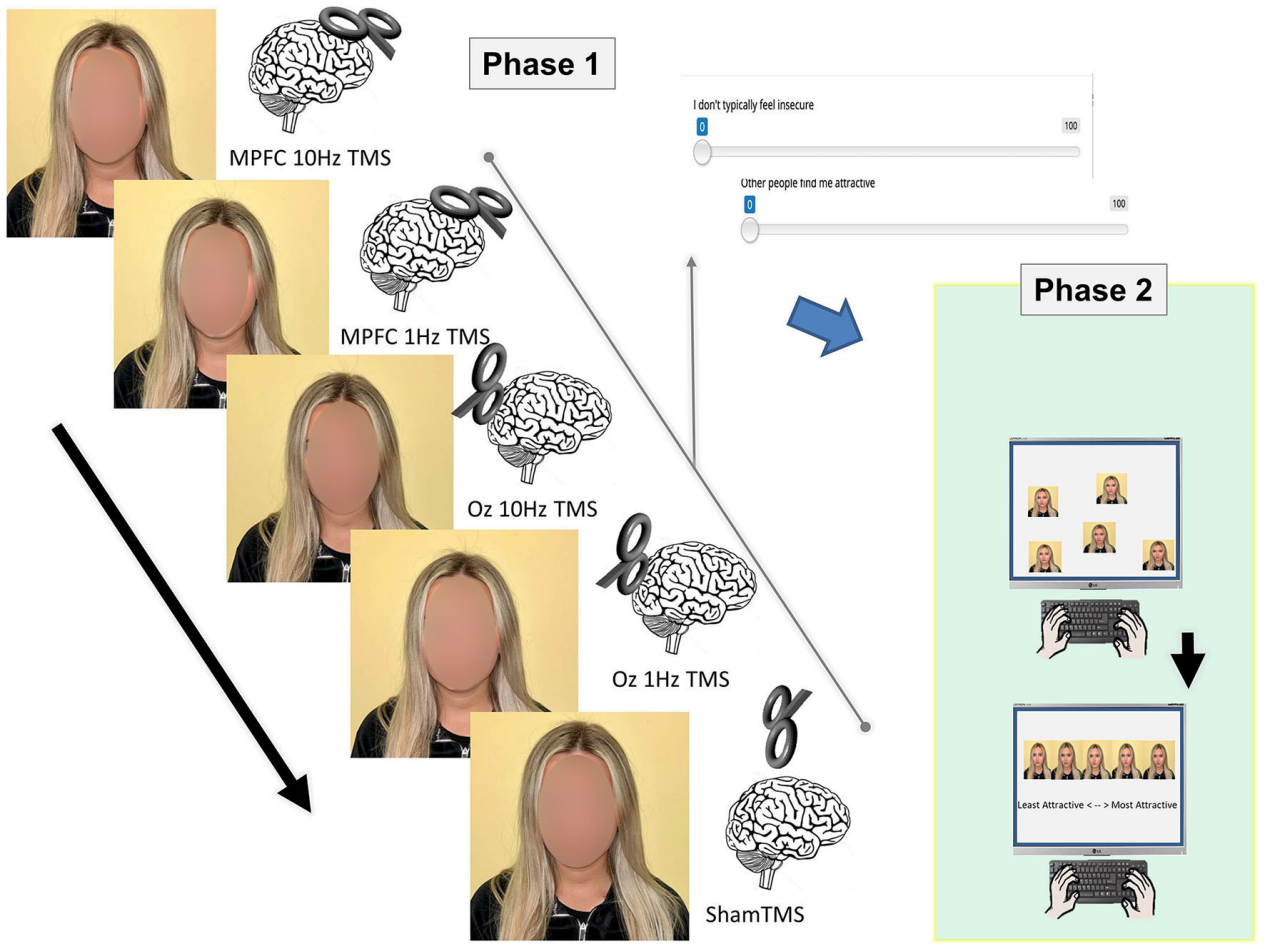
Two phases of the experiment. In Phase I, stimuli were prepared (i.e., photographs were taken) and self-ratings were made following TMS delivered to various regions (MPFC, OZ, and sham) and at different levels (10Hz and 1Hz). The photographs from Phase I were used in Phase II, where participants arranged the pictures from least attractive to most attractive.

For the stimuli for Phase I, photographs were taken, and self-attractiveness ratings were made after each of the 5 TMS sessions (see below). After each session, self-ratings were also made using a brief survey via Testable (Testable.org), totaling 23 statements (see Appendix). When the survey was conducted, answers were recorded using a slider bar that ranged from one to 100, with the numbers in between being hidden. The slider bar automatically began at 50 and could be manipulated lower or higher depending on how much the participant agreed or disagreed with the statement given (Figure 1).

For Phase II, the stimuli were produced by taking a total of five photos of each of the 10 participants, totaling 50 photos (from Phase I). In Phase II, photographs were randomly arranged and answers were recorded using drag-and-drop responses that included ratings of least attractive, second to least attractive, neutral, second to most attractive, and attractive.

### 2.4 Procedure

Prior to Phase I, each participant signed an informed consent form. To determine each participant's limit of stimulation, we adhered to Wasserman's and others' guidelines (Pascual-Leone et al., 1993; Wassermann, 1998; Lerner et al., 2019). While receiving TMS, each subject wore earplugs and a swim hat made of Lycra. Prior to starting TMS, the MPFC and Oz were both measured and marked on the swim cap.

To ensure that the proper level of TMS was applied, the participant's MT was established before the experiment started. The investigator applied supra-threshold TMS pulses to the contralateral abductor pollicis brevis muscle as each subject sat in a chair with their left hand outstretched. This revealed the location of the strongest motor-evoked potential (MEP) response. The TMS coil was then moved around the participant's scalp while being held at ~45° from the hemispheric line until the location that produced the greatest peak-to-peak amplitude MEP. Once an MEP of >50 μV was elicited after 50% of the TMS pulses had been delivered, the participant's MT was identified. The methods recommended by the International Federation of Clinical Neurophysiology were used to arrive at this conclusion. rTMS was administered throughout the experiment at 90% MT. All MT measurements were made via Trigno/DelSys.

rTMS was initiated after the participant's MT was determined. The MPFC and Oz were the regions of interest during rTMS. Each participant underwent five TMS conditions at 90% MT: (1) sham TMS, (2) 10 MPFC Hz rTMS, (3) 10 Oz Hz rTMS, (4) 1 Hz MPFC TMS, and (5) 1 Hz Oz TMS. To take into account the baseline (control) condition, sham TMS was conducted. The figure-of-eight TMS coil was held at a 90° angle over the vertex (typical 10/20 system coordinates) during the sham TMS. Sham was used as a placebo. A total of 300 pulses of TMS at 10 Hz were administered over five trains for 6 s. Each train was separated by a 20-s pause. A total of 300 pulses of TMS at 1 Hz were administered in one train over 5 min. For each subject, the TMS trials were performed in a different order. After each TMS session, the individual finished the 23-statement stimulus on Testable. The individual completed the stimulus a total of five times during the course of the experiment's five TMS trials.

For Phase II of the experiment, the participants recruited through SONA, rated each of the five photos of the 10 participants, resulting in the individuals completing 10 sets of ratings. No TMS was required for this phase of the experiment; the participants in Phase II were not aware of the TMS that took place in Phase I of the experiment. The participants were presented with the following instructions: “You will be given a series of five photographs of ten different people to look at as well as a set of labels. There are five labels consisting of: least attractive, second to least attractive, neutral, second to most attractive, and most attractive. You must drag each label to the picture that corresponds to said label. This is opinion based; there are no right or wrong answers so please label according to how you feel.”

### 2.5 Data analysis

In Phase I, the participants who received TMS completed the 23-question survey following TMS. A repeated measures ANOVA across the averaged 23 questions was performed with all of the questions averaged. We looked at the overall survey average as well as individual questions. We examined responses as well as reaction times to each question while completing the survey. By analyzing each question individually, the Type I error rate increased. Therefore, Bonferroni *post-hoc* corrections were planned and applied.

In Phase II, we used both parametric and non-parametric tests to ensure the validity of the findings. The rTMS condition was the main IV, with five levels. Both ANOVAs and the chi-squared test were corrected, again using the Bonferroni corrections.

## 3 Results

### 3.1 Phase I

It was found that there was no significant difference between any of the brain conditions [*F*_(1, 8)_ = 0.812, *P* > 0.05]. These data indicate that participants did not notice any change in their attractiveness or confidence across the brain conditions. Because the overall main effect was not significant, we did not perform *post-hoc* tests.

**Figure 2:**
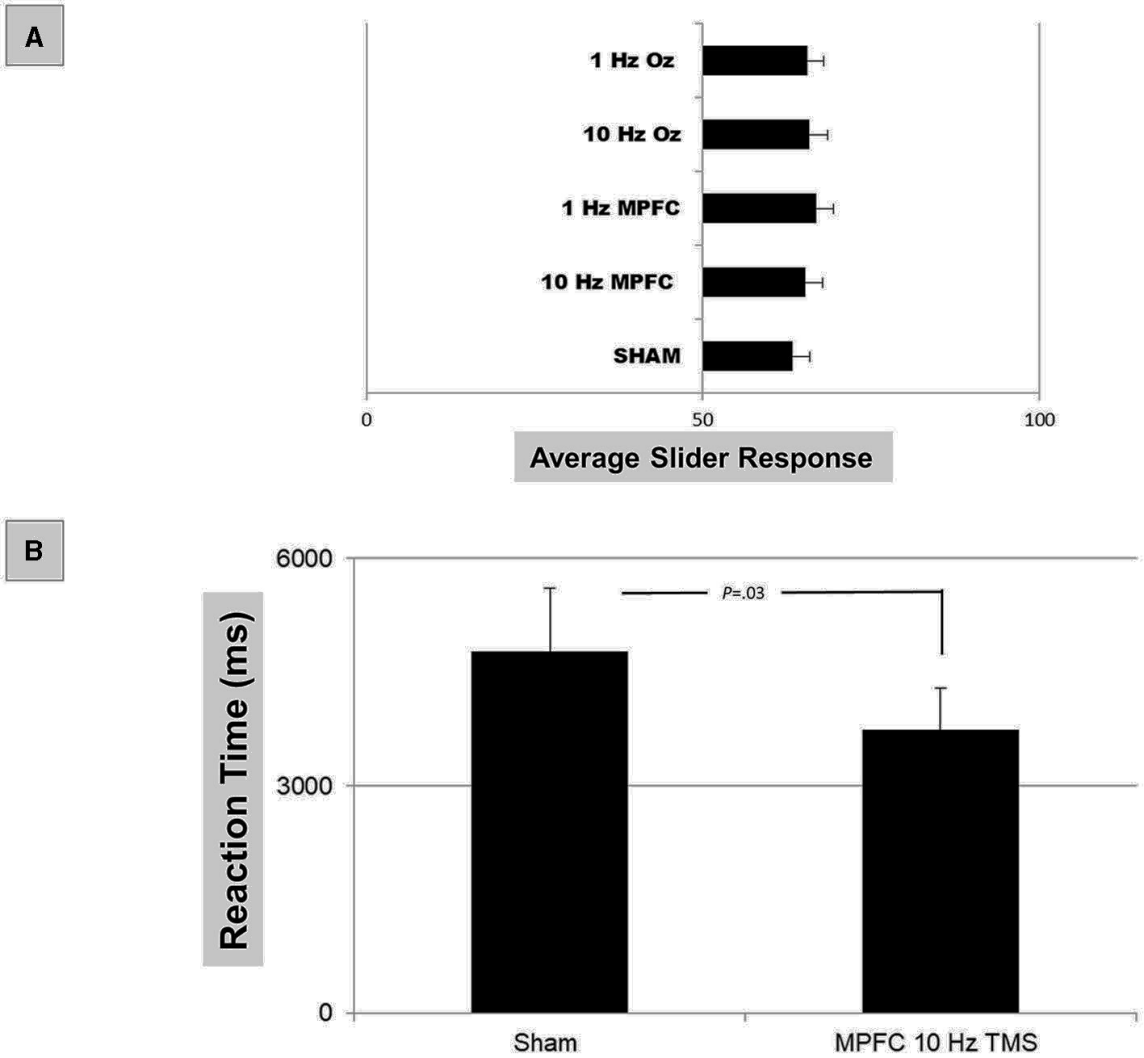
Shown are the results of the Testable provided average responses in Phase I of the experiment **(A)**. In terms of response, there was no overall significance (all *p* > 0.05). When analyzed at an individual question level, reaction time **(B)** was quicker for the question “I am Attractive” when non-corrected (*p* = 0.03), but it did not survive Bonferroni corrections (*p* > 0.05). We therefore conclude that no differences existed in self-ratings.

We then looked at self-rated individual questions in terms of response. The main question, ‘I am attractive' was non-significant [*F*_(4, 28)_ = 0.192, *p* = 0.94) when compared across the brain conditions, indicating a person did not report feeling any different in terms of their attractiveness (Figure 2). In fact, there were no differences between any of the individual questions (all *p*> 0.05) in terms of response. ‘I am attractive', however, was significantly different in terms of reaction time after MPFC 10 Hz TMS (*M* = 3740.4, SE = 545.11) when compared to sham [*M* = 4773.8, SE = 839.17; *t*(9) = −2.09, *p* = 0.03]. These data indicate that following MPFC 10Hz, participants were quick to respond that they were attractive, perhaps indicating that they were consciously aware of their appearance. However, when Bonferroni corrections were applied, this difference was non-significant. No other reaction times were different across any of the questions or TMS conditions.

### 3.2 Phase II

**Figure 3:**
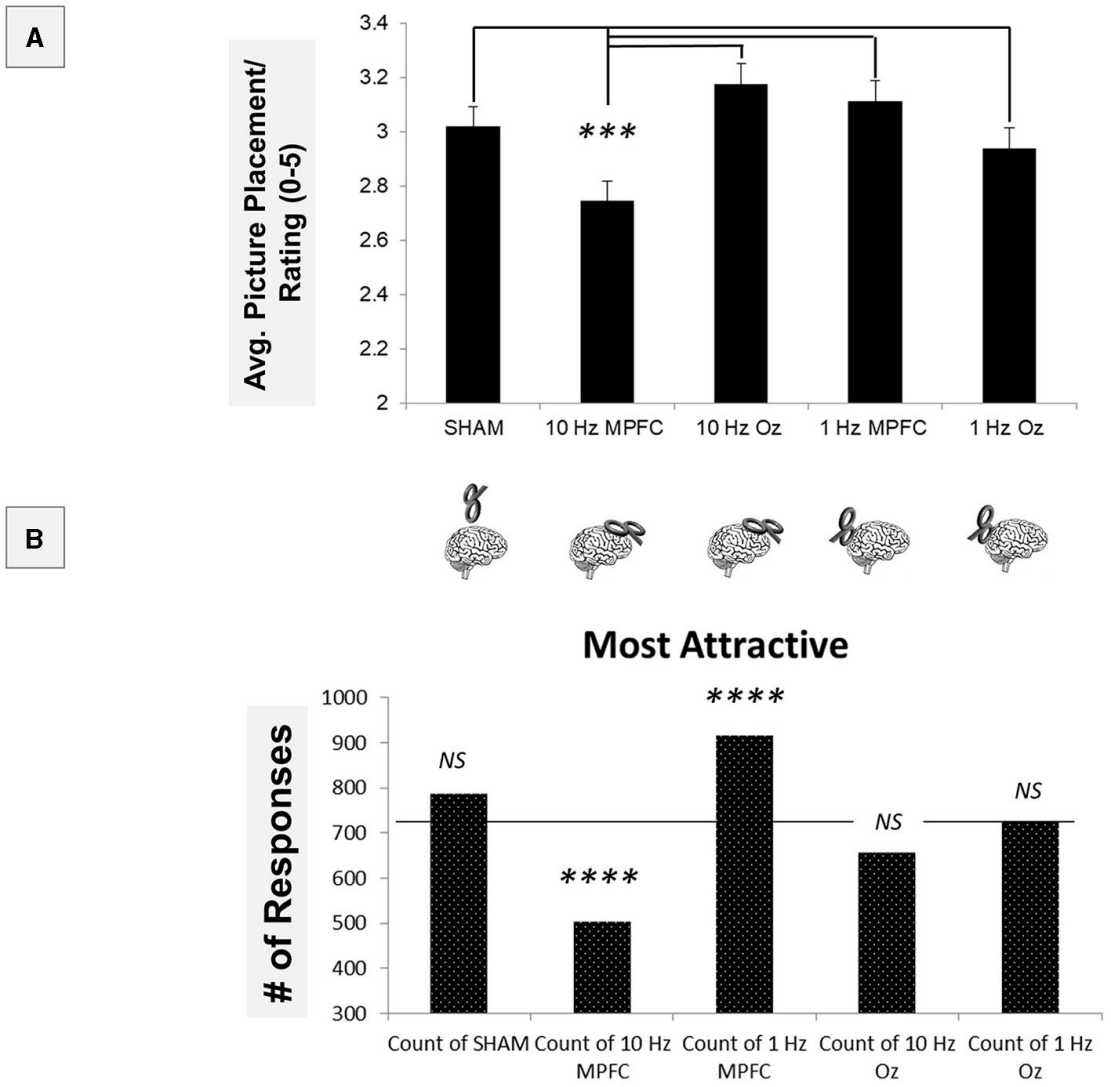
Using parametric statistics, it was found that 10Hz MPFC TMS resulted in significant differences when compared to all other conditions **(A)**. To determine other differences across non-parametric measures, “Most Attractive” ratings were compared across the TMS conditions **(B)**. Both 10Hz and 1Hz MPFC TMS resulted in significant differences compared to the average response. ***p < 0.001; ****p < 0.00001.

We averaged each of the 10 rated participants into a single score and performed a repeated measures ANOVA. It was found that there was an overall significant difference: [*F*_(4, 1468)_ = 39.454, *p* < 0.001, see Figure 3A]. The *post-hoc* test revealed that 10 Hz MPFC stimulation resulted in significantly lower attractiveness ratings compared to all other conditions (all *p* < 0.001). These data indicate inhibiting the MPFC increases attractiveness. All other contrasts were non-significant (all *p* > 0.05).

To follow up on this, we performed a series of non-parametric tests, first employing a 5 × 5 in which rating (“most attractive” to “least attractive”) was compared across the TMS brain site (Figure 3B). The 5x5 chi-square revealed a significant overall effect [*X*^2^(16) = 688.21, *p* < 0.00001]. This indicated that ratings varied across TMS sites. Using adjusted the Bonferroni comparisons (*p* < 0.001), we examined ‘Most Attractive' ratings. Compared to the average rating, it was found that 10 Hz MPFC significantly reduced the number of ‘Most Attractive' selections [*X*^2^(1) = 63.61, *p* < 0.00001].

Furthermore, within the MPFC, 1 Hz MPFC significantly increased the number of selections [*X*^2^(1) = 57.8, *p* < 0.00001]. Sham [*X*^2^(1) = 6.42, *p* > 0.001], 10Hz Oz [*X*^2^(1) = 5.34, *p* > 0.001], and 1 Hz Oz [*X*^2^(1) = 0.05, *p* > 0.001] were not significant. These data indicated that 10Hz TMS decreased attractiveness, while 1Hz TMS increased attractiveness.

## 4 Discussion

This study sought to investigate the potential effects of TMS on attractiveness in the way another person perceives an individual. For Phase I of the experiment, we found an overall lack of change in self-rated feelings of attractiveness. Phase II of the study revealed that increases in MPFC activity decreased attraction, while decreased activity increased the rated attractiveness of the participants. To our knowledge, this is the first study to demonstrate that direct manipulation of the brain via TMS alters a person's attractiveness.

Prior research has suggested a link between how faces are perceived and the MPFC (Richardson et al., 2021), possibly due to its role in recognizing facial expressions and processing emotions. However, it is possible that overconfidence, below the level of awareness of the stimulated participants, may cause attractiveness to decrease (Zeigler-Hill and Myers, 2011), which may explain why inhibitory TMS to the MPFC resulted in a “more attractive” rating and excitatory TMS decreased attraction. Future studies should further investigate inhibitory TMS to the MPFC vs. excitatory TMS to the MPFC and how it relates to overconfidence and attractiveness. That being said, the data are quite consistent with previous research in which MPFC TMS (Barrios et al., 2008; Amati et al., 2010; Taylor-Lillquist et al., 2020; Duran et al., 2021) and other default mode network regions (Luber et al., 2012) impact confidence and overconfidence. Therefore, we believe that confidence may have been altered at a level below the perception of the subjects whose brains were stimulated. Unfortunately, our study did not examine this possibility, and further research is needed.

Our data are comparable to those of Karamann. They found that 1Hz rTMS increased perceived pain responses. We interpret our findings similarly, in that changes in facial expression were caused directly by the dysregulation of the MPFC. Such dysregulation of the MPFC reduces an individual's ability to monitor and/or inhibit facial microexpressions. Therefore, emotions are expressed more directly following 1Hz rTMS. These data are further supported by studies on infants. Early in life, facial expressions are less regulated and more stimulus-bound. As the MPFC develops and emotion regulation increases, facial expression also becomes more regulated (Saarni, 1984). We believe here that a more expressive face is seen as more attractive (Rennels and Kayl, 2015).

Although these findings may suggest a relationship between attractiveness and the MPFC, there were certain limitations to the experiment. The Phase I sample size of 10 was modest, though adequate power was achieved (confirmed by the significant findings in Phase II; see also (Taylor-Lillquist et al., 2020) using methods that we have previously used (Shelansky et al., 2022). Our participants were undergraduates, and our results are limited in how they should be applied to a general population. We also acknowledge that we were not monitoring SONA participants directly, as is the case with the online use of an experimental subject pool. The benefit of SONA relates to the previous point in that it is rare to have rTMS studies also include such a large sample. This novel approach, we hope, finds traction in the literature. Beyond the scope of this paper was emotion, such as empathy (Yoder et al., 2015; Decety et al., 2016) and its role in the brain (Kim and Hamann, 2007), which plays a role in most higher-order selections and is associated with frontal and cingulate regions (Shackman and Wager, 2019a,b; Hur et al., 2022). That said, a number of the questions in Phase I tapped into the theory of mind, and there were no significant differences found. Clearly, future studies should examine raters' empathy and the theory of mind. Furthermore, while gender/sex plays a role in attraction (Hamann et al., 2004; Stevens and Hamann, 2012), we expressly avoided this in terms of recruiting and testing/questions. We think that there may be a role here, but we attempted to avoid explicit sexual attraction.

We suspect that these data encourage much in terms of future research. For example, we would want to see ratings based on actual interactions rather than photographs. Furthermore, those doing the ratings should be interviewed following the study to determine why they found certain versions of a person more attractive. Finally, we hope to use a smaller rater sample and perform neuroimaging to determine subtle (we assume) brain changes that might be associated with changes in the raters' choices.

In conclusion, the perception of an individual's attractiveness may be influenced after an individual receives TMS from the MPFC. These data support the notion that the dysregulation of the MPFC influences the facial musculature such that a person's attractiveness is altered, which may reflect an underlying change in cognitive state. As with previous findings, these changes occur below the level of one's own perceptual awareness. The role of the MPFC in facial perception may be better understood in light of these findings.

## Data availability statement

The raw data supporting the conclusions of this article will be made available by the authors, without undue reservation.

## Ethics statement

The studies involving humans were approved by the Montclair State University IRB. The studies were conducted in accordance with the local legislation and institutional requirements. The participants provided their written informed consent to participate in this study.

## Author contributions

SZ: Conceptualization, Data curation, Investigation, Visualization, Writing – original draft, Writing – review & editing. CS: Investigation, Methodology, Project administration, Writing – original draft. SA: Writing – review & editing. MS: Investigation, Methodology, Writing – review & editing. AM: Investigation, Methodology, Writing – original draft. AL: Formal analysis, Investigation, Writing – original draft. MP: Investigation, Methodology, Project administration, Writing – original draft. JK: Conceptualization, Data curation, Formal analysis, Funding acquisition, Investigation, Resources, Supervision, Writing – original draft, Writing – review & editing.

## Appendix

**Table A1:** Statements included in stimuli presented to participants.

**Statement**
I am confident
I am intelligent
I am attractive
Other people find me confident
Other people find me intelligent
Other people find me attractive
I never make mistakes
I have a lot of friends
My friends think highly of me
I am often correct
I don't typically feel insecure
People give me their attention wherever I go
I always look put together
I am talented
I am myself around everyone I meet
People often come to me for advice
I don't typically judge people
I get asked out often
I am a nice person
I go out of my way to help people
I am a patient person
I am respectful
I am humble
